# Distinct Microtubule Organizing Center Mechanisms Combine to Generate Neuron Polarity and Arbor Complexity

**DOI:** 10.3389/fncel.2020.594199

**Published:** 2020-11-19

**Authors:** Oliver R. Wilkes, Adrian W. Moore

**Affiliations:** ^1^Laboratory for Neurodiversity, RIKEN Center for Brain Science, Wako-shi, Japan; ^2^Department of Cellular and Molecular Biology, Institute for Translational Medicine, University of Liverpool, Liverpool, United Kingdom

**Keywords:** microtubule nucleation, microtubule organization, neuron differentiation, axon, dendrite

## Abstract

Dendrite and axon arbor wiring patterns determine the connectivity and computational characteristics of a neuron. The identities of these dendrite and axon arbors are created by differential polarization of their microtubule arrays, and their complexity and pattern are generated by the extension and organization of these arrays. We describe how several molecularly distinct microtubule organizing center (MTOC) mechanisms function during neuron differentiation to generate and arrange dendrite and axon microtubules. The temporal and spatial organization of these MTOCs generates, patterns, and diversifies arbor wiring.

## Introduction

Nervous system computation requires intricate neuronal wiring patterns. To achieve this, a differentiating neuron generates complex axon and dendrite arbors. The generation of these arbors depends upon concurrent construction of specialized microtubule networks within the extending and branching dendrites and axons. The invasion of polymerizing microtubules into the tips of elongating axons and dendrites exerts a growth force (Kapitein and Hoogenraad, 2015; Santos et al., 2020), and it provides tracks for microtubule motors to traffic machinery and materials for further growth (Schelski and Bradke, 2017; Burute and Kapitein, 2019; Kelliher et al., 2019).

It is well-described that within the neuron a suite of microtubule associated proteins (MAPs) and microtubule associated motors organize the microtubules, and that these microtubules undergo post-translational modifications that alter their stability and their interactions with molecular motors; for recent reviews please see (Kapitein and Hoogenraad, 2015; Lasser et al., 2018; Park and Roll-Mecak, 2018; Burute and Kapitein, 2019; Kelliher et al., 2019). On the other hand, despite a clear necessity for local microtubule generation during neuron differentiation, the underlying mechanisms have remained unclear.

At the heart of these mechanisms is *de novo* microtubule generation—the process where new microtubules are nucleated within the cell. As it is kinetically unfavorable to nucleate microtubules by spontaneously arranging free heterodimers, cellular mechanisms favor the construction of short microtubule seeds from which further polymerization can proceed. This seed formation is organized spatially within the cell, and it is often concentrated at microtubule organizing centers (MTOCs). Although the mechanisms by which MTOCs template and arrange microtubules into high-order structures in proliferating cells are well-documented (Prosser and Pelletier, 2017; Martin and Akhmanova, 2018; Paz and Luders, 2018), how this occurs in postmitotic neurons is only now being understood.

In this article, we summarize recent data that reveal several distinct modes of MTOC formation and *de novo* microtubule generation within a differentiating neuron. Because final complex arbor wiring patterns are generated through a series of morphogenetic differentiation events (Yoong et al., 2019), we then go beyond the level of molecular mechanism to synthesize a view at the systems level. We discuss how the usage of distinct microtubule generation mechanisms changes as arbor formation proceeds, how they are spatially and temporally organized in the differentiating neuron to generate arbor complexity, and how changing the relative activity of distinct MTOC mechanisms diversifies arbor patterning.

## Organization and Competition Between Molecular Machineries of *De Novo* Microtubule Generation

Microtubule seed generation occurs via the γ-Tubulin ring complex (γ-TuRC). This complex is formed from γ-Tubulin (γ-Tub) and the Tubulin gamma complex associated proteins (Tubgcp) 2–5, in vertebrates (Grip75, 84, 91, 128, and 163 in *Drosophila*). Tubgcp 2–5 assemble into an inverted cone (Consolati et al., 2020; Liu et al., 2020; Wieczorek et al., 2020). In purified human γTuRC shows, this was shown to be further stabilized by MITOTIC SPINDLE-ORGANIZING PROTEIN 2 (MZT2); (Consolati et al., 2020). γ-Tub monomers assemble into a ring atop the inverted cone. Each γ-Tub monomer serves as a surface for the β face of the initial α/β-Tub heterodimer to bind, with the ring structure as a template for the tubular organization of the resultant microtubule. Individual filaments extend by progressive end-to-end stacking of α/β-Tub heterodimers. At the same time, neighboring filaments bind together to create the characteristic tubular organization. The exposed α-Tub face is called the plus-end, and growth initiates from this face at the seed stage. Plus-ends of microtubules are fast-growing and represent the major sites of microtubule elongation in cells, including neurons (Feng et al., 2019). The β face, which is attached to the to γ-TuRC at the seed stage, is the minus-end. This often stays attached to the γ-TuRC to prevent depolymerization. However, if the γ-TuRC is removed, the exposed β face is instead capped with an alternative complex which, in neurons, has been shown to be through Calmodulin-regulated spectrin-associated protein (Camsap in mammals, Patronin in *Drosophila*) family members (Yau et al., 2014; Feng et al., 2019). This Patronin cap stabilizes the minus-ends; it also promotes slow minus-end polymerization (Martin and Akhmanova, 2018; Akhmanova and Steinmetz, 2019).

In addition, assembly of a microtubule seed is catalyzed by Tpx2 Targeting Protein for Xklp2 (Tpx2 in vertebrates; Mei38 in *Drosophila*) and Cytoskeleton associated protein 5 (Ckap5; also called Xmap215, or Colonic and hepatic tumor overexpressed gene protein (Ch-TOG) in vertebrates; Minispindles [msps] in *Drosophila*). Tpx2 acts by binding across neighboring longitudinal and lateral tubulin dimers (Li et al., 2017), while Ckap5 binds to a single γ-Tubulin monomer at the γ-TuRC and extends out to recruit and stabilize a line of α/β-Tubulin heterodimers along the seed filament (Thawani et al., 2018). Notably, through linear heterodimer binding action, Ckap5 also continues to promote polymerization of *bone fide* microtubule beyond the initial stage of seeding (Thawani et al., 2018). Both these factors also catalyze microtubule formation in the absence of γ-TuRC (Wieczorek et al., 2015).

γ-TuRCs are usually recruited to MTOCs, where they are arranged and activated to give rise to microtubules. In dividing cells, the centrosome, mitotic spindle, and Golgi stacks are well-described sites of MTOC activity. Recruitment to MTOCs is managed by a suite of γ-TuRC-tethering proteins (γ-TuRC-TPs); these include Neural precursor cell Expressed, Developmentally Down-regulated protein 1 (Nedd1 in vertebrates; Grip71 in *Drosophila*), Mitotic Spindle Organizing Protein 1 (Mzt1), CDK5 Regulatory Subunit Associated Protein 2 (Cdk5rap2 in vertebrates; Centrosomin [Cnn] in *Drosophila*), Myomegalin, and Pericentrin (Pcnt in vertebrates; Pericentrin like protein [Plp] in Drosophila). An overview of γ-TuRC-TP structure and mechanism can be found in (Tovey and Conduit, 2018). A subset of these γ-TuRC-TPs, Cdk5rap2/Cnn, Myomegalin, and Pcnt (but not Plp), contain a CM1 domain (Centrosomin1 domain) which activates γ-TuRC to induce production of microtubules (Choi et al., 2010). This activation event is likely through a conformational change in γ-TuRC (Choi et al., 2010; Consolati et al., 2020; Liu et al., 2020; Wieczorek et al., 2020).

Overall, distinct MTOC activities are created by using characteristic compositions of γ-TuRC-TPs and employing these γ-TuRC-TPs in specific phosphorylation states. In dividing cells, the centrosome serves as the principal MTOC. It is surrounded by a network of γ-TuRC-TPs including Pcnt, Cdk5rap2, and Nedd1, and scaffolding factors including Ninenin (Nin) and Centrosome and Golgi localized protein kinase N (PKN)-associated protein (CG-NAP; also called A-kinase anchoring protein 450 [AKAP450]) in mammals. Together, these make up the pericentriolar material (PCM), which recruits and activates γ-TuRC to nucleate the mitotic spindle during cell division. In addition, acentrosomal nucleation events also occur to further build the spindle. One such event utilizes a RanGTP (Ras-related nuclear protein) gradient to trigger nucleation and promote the interaction of the γ-TuRC complex with Tpx2 in a process that is regulated by Aurora A kinase phosphorylation (Pinyol et al., 2013). Another event involves the eight proteins of the Augmin complex [known as homologous to Augmin subunits [HAUS] in vertebrates (Goshima et al., 2008; Lawo et al., 2009)]. Augmin targets γ-TuRC onto an existing host microtubule. It initiates a nucleation event that forms a new microtubule from the side of the pre-existing host microtubule; this new microtubule inherits the same polarity within the cell as the host (Kamasaki et al., 2013; Petry et al., 2013). For detailed reviews of these MTOC mechanisms see (Prosser and Pelletier, 2017; Martin and Akhmanova, 2018; Paz and Luders, 2018; Akhmanova and Steinmetz, 2019).

MTOCs compete amongst themselves for common resources within a cell; for example, in *Drosophila* syncytial embryos, blocking Augmin-dependent nucleation in spindles increases centrosome activity, while reducing centrosomal nucleation activity increases nucleation in the spindle (Hayward et al., 2014). A tug-of-war like competition for a restricted population of γ-TuRC recruitment factors is the likely mechanism [further discussed by (Tann and Moore, 2019)]. Because of this potential for tug-of-war competition between MTOCs, one way to shape the microtubule network during neuron differentiation is by differential regulation of γ-TuRC-TPs levels or activity, and an example of this is neuron type-specific control of Cnn in *Drosophila* da neurons (Yalgin et al., 2015).

The fundamental MTOC components described in other cell types are similarly utilized in neurons to generate and organize microtubule networks during arbor differentiation. Experimental manipulations of γ-Tub or γ-TuRC-TPs in neurons lead to a series of changes in microtubule density, organization, and polarity orientations in the axon, dendrites, and soma; in addition, these changes often alter arbor patterning (Ori-McKenney et al., 2012; Nguyen et al., 2014; Zhou et al., 2014; Yalgin et al., 2015; Sánchez-Huertas et al., 2016; Cunha-Ferreira et al., 2018; Liang et al., 2020; Weiner et al., 2020). As we describe below, during neuron differentiation, these components are organized into a series of different MTOC forms.

## Neuronal Precursor Cell Mitotic Machinery Is Reutilized in the Nascent Neuron to Support Initial Neurite Outgrowth

The generation of microtubule networks in a differentiating neuron is aided by a series of MTOCs, the composition and organization of which evolve throughout the process. Initially, the neuron inherits a centrosome from its mother, and at the first stages of differentiation in cultured rodent hippocampal, cortical, and dorsal root ganglion (DRG) neurons, the centrosome continues to nucleate microtubules (Mori et al., 2009; Stiess et al., 2010; Rao et al., 2016; Yamada and Hayashi, 2019). On the other hand, this is not seen in *Drosophila* sensory neurons *in vivo* (Nguyen et al., 2011). Some of these microtubules are transported into neurites via motor-based microtubule sliding to support initial outgrowth that provides both plus-ends-out and minus-ends-out microtubule populations; see reviews by (Baas and Falnikar, 2012; Del Castillo et al., 2019).

**FIGURE 1:**
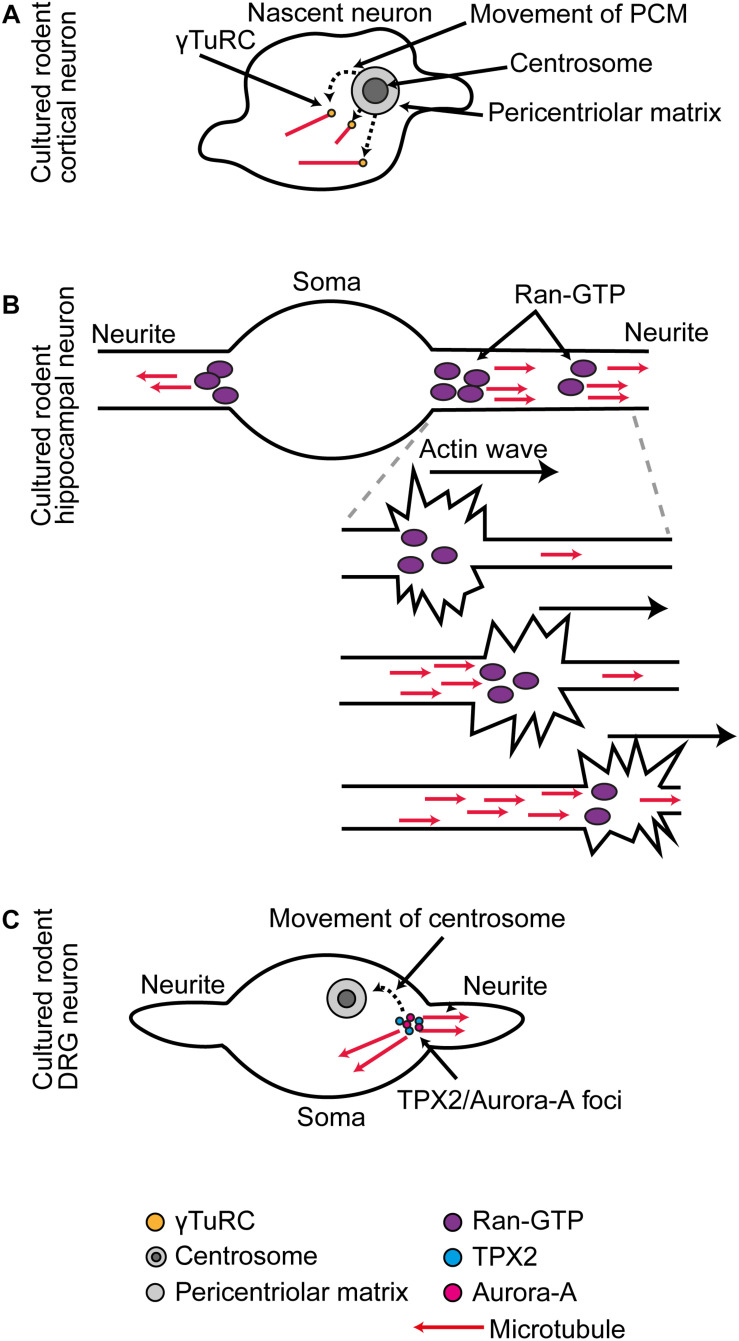
**(A)** In cultured rodent cortical neurons, as the centrosome is decommissioned as an MTOC, there is a phase of somatic γ-Tub-mediated microtubule organization. **(B)** In cultured rodent hippocampal neurons, RanGTP localization at the base and distal domains of the extending neurite supports Tpx2-mediated microtubule generation. During neurite outgrowth actin waves that progress along the neurites trigger increased microtubule generation in their wake. **(C)** In cultured rodent DRG neurons, the centrosome is first situated at the base of one neurite, and at this position Tpx2 facilitates Aurora A activation. As the centrosome migrates away, it leaves behind a new Aurora A-Tpx2 based MTOC.

Then, the centrosome loses MTOC activity. The primary driver for this change is likely to be the post-mitotic downregulation of Nedd1 (Stiess et al., 2010; Sánchez-Huertas et al., 2016) and alternative splicing of Nin (Srivatsa et al., 2015; Zhang et al., 2016), as these changes are observed occurring in parallel with the loss of centrosome MTOC activity in rodent hippocampal and cortical cultures. γ-Tub, Pcnt, Cdk5rap2, Nin, Tpx2, and Nedd1 are all reduced or lost from the centrosome and shifted to new sites (Baird et al., 2004; Mori et al., 2009; Ohama and Hayashi, 2009; Stiess et al., 2010; Srivatsa et al., 2015; Yonezawa et al., 2015; Sánchez-Huertas et al., 2016; Zhang et al., 2016). For example, in cultured rodent cortical neurons, after the PCM is dismantled, there is a transient period in which γ-Tub/Mzt2 positive puncta spread throughout the soma, giving rise to microtubules (Yamada and Hayashi, 2019; Figure 1A).

In cultured rodent hippocampal neurons, RanGTP becomes concentrated in two positions in every newly forming neurite: one at the base and one in the distal portion. At these positions, RanGTP triggers Tpx2-dependent microtubule generation (Chen et al., 2017; Figure 1B). In cultured rodent DRG neurons, there is a local hand-off of Tpx2-centered nucleation machinery from the centrosome to an acentrosomal site. Initially, the centrosome is located at the base of one neurite, where it generates microtubules. Then, the centrosome migrates away from this position and at the same time it stops generating microtubules. When this occurs, it leaves behind a local foci of Aurora A kinase and Tpx2 that continues as a new MTOC (Mori et al., 2009; Figure 1C).

Microtubule generation within neurites is also triggered concomitant with the passage of actin waves. These are traveling waves of transient local actin reorganization into filopodia and lamellipodia that move slowly along the neurite from its base to its tip (Flynn et al., 2009). In cultured rodent hippocampal neurons, a local increase in microtubule generation activity occurs in the wake of the wave (Winans et al., 2016; Figure 1B). It is possible that RanGTP-Tpx2 based mechanisms are responsible for this local amplification of microtubule generation as actin waves transport RanGTP (Huang et al., 2020; Figure 1B).

## Maintenance of Axon Microtubule Cytoskeleton Unipolar Organization

Alongside neurite formation, nascent neurons must polarize (Schelski and Bradke, 2017; Yogev and Shen, 2017). This is usually into one axon and multiple dendrites, although some specialized neuron types develop other configurations (Troutt et al., 1990; Mori et al., 2009; Harterink et al., 2018). Microtubules in the axon are predominantly oriented plus-ends-out, an anterograde organization (Baas et al., 1988; Yau et al., 2016). While in the dendrites of vertebrate neurons microtubules are a mix of minus-ends-out (retrograde) and plus-ends-out orientations (Baas et al., 1988; Kleele et al., 2014; Yau et al., 2016), in the dendrites of invertebrate neurons (demonstrated in *Drosophila* and *C. elegans*), they are predominantly minus-ends-out (Rolls et al., 2007; Goodwin et al., 2012). Importantly, these organizational differences of microtubule polarity direct compartment-specific trafficking of cargo within the neuron (Burute and Kapitein, 2019; Kelliher et al., 2019).

A complex set of signaling events are used to select one neurite to become the axon (Schelski and Bradke, 2017; Yogev and Shen, 2017). Even so, as demonstrated in cultured rodent hippocampal neurons, at the point when one neurite becomes the axon it shows a selective enhancement of stable plus-ends-out microtubules (Witte et al., 2008; Yau et al., 2016). One process involved in generating and maintaining axon unipolar organization is microtubule sliding (Baas and Falnikar, 2012; Del Castillo et al., 2019). A second is microtubule generation through Augmin.

**FIGURE 2:**
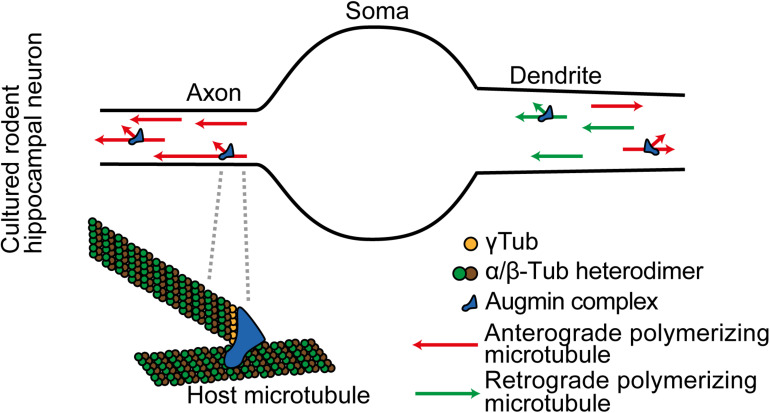
In cultured rodent cortical neurons, Augmin complexes are localized together with γ-TuRC throughout differentiating axons and dendrites. They locally amplify microtubule generation. Augmin also supports plus-ends-out microtubule generation in the axon.

Knockdown of Augmin components impedes cortical neuron polarization *in vivo*, and it suppresses the ability of drug-mediated microtubule stabilization to induce supernumerary axons in cultured rodent hippocampal neurons (Sánchez-Huertas et al., 2016; Cunha-Ferreira et al., 2018). In the axon, while there is evidence of a proximal enriched region of microtubule generation in both cultured rodent hippocampal neurons and multiple *C. elegans* neuron types *in vivo* (Yau et al., 2014; Harterink et al., 2018), data from the cultured rodent hippocampal neuron model shows that Augmin generates microtubules along the axon length. Similarly, it generates microtubules throughout the dendrites (Sánchez-Huertas et al., 2016; Cunha-Ferreira et al., 2018; Figure 2).

Augmin plays a further specific role in the axon; it maintains the specialized unipolar organization of axon microtubules. A key aspect of Augmin activity described in the spindle of human U2OS cells and in meiotic Xenopus egg extracts is that it nucleates a new microtubule that polymerizes with the same polarity as the host microtubule upon which it was initiated (Kamasaki et al., 2013; Petry et al., 2013). It is expected that the same mechanism occurs in neurons, and this explains changes in microtubule polarities in the axon when Augmin activity is lost. In the axon all the potential host microtubules are plus-ends-out, and Augmin activity enables newly generated microtubules to maintain this unipolar organization (Figure 2). With loss of Augmin, the new microtubules that form grow in either direction (Sánchez-Huertas et al., 2016; Cunha-Ferreira et al., 2018; Qu et al., 2019).

## MTOCs Track the Growing Tips of Primary Dendrites

In contrast to the axon, dendrite differentiation requires the generation of both plus-ends-out and minus-ends-out microtubules. Two recent studies in *C. elegans* PVD and *Drosophila* da neurons show how MTOCs localized in the dendritic growth cones generate both a minus-ends-out and a plus-ends-out population that invade into the growing dendrite tip (Liang et al., 2020; Yoong et al., 2020).

**FIGURE 3:**
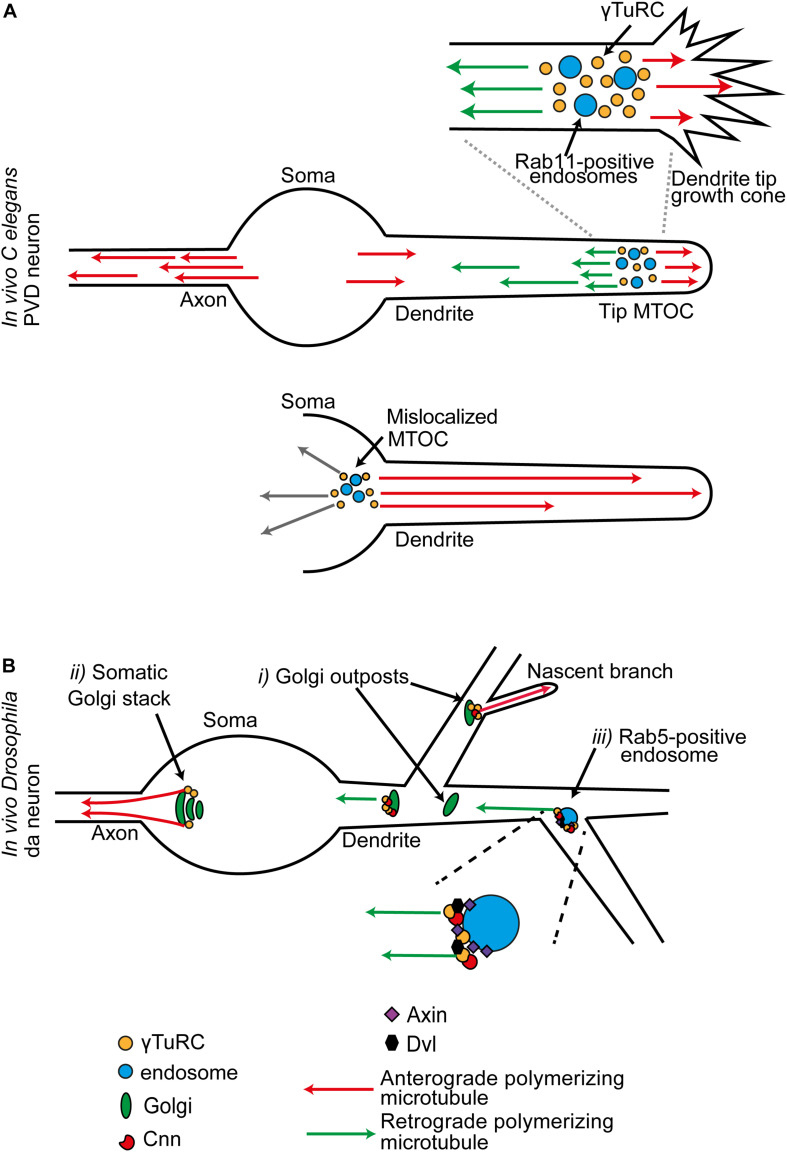
**(A)** In *C. elegans* PVD neurons, dendritic growth cone MTOCs give rise to two distinct populations of polymerizing microtubules: plus-ends-out microtubules that invade the tip, and minus-ends-out microtubules that create the specialist polarity organization of the dendrite. This MTOC consists of γ-TuRC organized around a population of RAB11-positive endosomes. When the MTOC is mislocalized to the soma, all microtubules in the dendrite are now plus-ends-out. **(B)** Sites of MTOC activity in late stage neurons, illustrated for the combined data from multiple studies in *Drosophila* da neurons. **(*i*)** A subset of Golgi outposts is associated with γ-Tub, and unidirectional microtubule generation is promoted from some. **(*ii*)** In the mature neuron, Golgi mediated microtubule generation is principally from stacks in the soma. These generate a population of polymerizing microtubules that exclusively invade the axon. Golgi may also act as local site of microtubule generation at the branchpoints of a nascent branch. **(*iii*)** γ-Tub is localized at Rab5-positive endosomes at dendrite branchpoints. γ-TuRC-TPs and components of the Wnt signaling pathway are required for microtubule generation activity at these positions.

In the dendritic growth cone of the *C. elegans* PVD neuron, γ-TuRCs assemble around RAB11-positive endosomes. This site at the tip of the extending dendrite is an MTOC that produces both anterograde and retrograde polymerizing microtubule populations; these create the plus-ends-out and minus-ends-out microtubule arrays of the dendrite, respectively (Figure 3A). Compared to the minus-ends-out population, the plus-ends-out microtubules that are generated from the tip MTOC pause longer between polymerization and depolymerization. When a sufficient plus-ends-out array is established, the MTOC moves toward the tip so that it tracks tip extension. With loss of the plus-end directed motor Kinesin-1 (UNC-116 in *C. elegans*), the MTOC is misplaced in the cell body; all microtubules in the dendrite now polymerize in from the soma, creating an axon-like unipolar plus-ends-out array (Figure 3A). Moreover, as Kinesin-1 prefers to move on stable microtubules, which are the plus end out population, these data suggest a model in which Kinesin-1 is engaged to move the MTOC along this plus-ends-out array so that it tracks tip extension (Liang et al., 2020).

A tip MTOC is also seen in *Drosophila* da neurons. During primary dendrite outgrowth, a network of actin regulators centered around the actin motor Myosin6 set both the position and direction of the microtubule polymerization events generated from a dendritic growth cone MTOC. Furthermore, this MTOC is utilized in the splitting of the tip into new primary branches. Splitting correlates with surges in the generation of the anterograde polymerizing population; these anterograde polymerizing microtubules are then guided into nascent branches via retrograde extension of actin filaments at the base of growth cone filopodia (Yoong et al., 2020).

The growth cone MTOC at the tip of a growing dendrite is a developmental structure required to create and organize the microtubules of the primary dendrite arbor branches; a different kind of tip MTOC is found in some specialized mature sensory neurons (Harterink et al., 2018). These sensory neuron types have a single dendrite tipped with a sensory cilium (Troutt et al., 1990; Harterink et al., 2018). At the base of cilia is a basal body, which is created from a centriole that is reutilized after the centrosome has been decommissioned, and imaging of differentiating *C. elegans* neurons showed how a centriole is trafficked from the soma to the dendrite tip (Li et al., 2017). γ-TuRC components localize at the base of the cilia, and this region acts as an MTOC to produce anterograde polymerizing microtubules. Multiple *C*. *elegans* neuron types were examined to study the functional output of having this MTOC. In *C*. *elegans* neuron types without a distal MTOC, microtubule motor-based cargo transport is more efficient in the proximal dendrite, but it drops off in the distal dendrite. In contrast, in those types with a dedicated MTOC at the base of the cilia, the transport remains efficient along the length of the dendrite (Harterink et al., 2018).

## MTOCs Associated With High Order Dendrite Branches

Dendritic growth cone MTOCs play a role in the formation of primary branch structure. However, neurons pattern through an evolving set of processes rather than repetitive use of a single set of local cell biological operations (Yoong et al., 2019, 2020). For the formation of high order branches, different processes are required. In multiple models including rodent hippocampal and cortical neurons, chick DRG neurons and *Drosophila* da neurons, high order branches form interstitial pioneer filopodia and lamellipodia that are then stabilized by the invasion of microtubules (Kalil and Dent, 2014).

In dendrites, microtubule invasion from the main dendrite trunk into higher order compartments occurs in differentiation processes, such the formation of terminal branches in *Drosophila* da neurons or spines in rodent hippocampal neuron cultures (Gu et al., 2008; Ori-McKenney et al., 2012; Stewart et al., 2012; Yalgin et al., 2015). It also occurs in activity-dependent spine remodeling in the mature neurons, as shown in rodent hippocampal neuron cultures and slice cultures (Hu et al., 2008; Jaworski et al., 2009; Merriam et al., 2011; Schatzle et al., 2018). This activity-dependent invasion of microtubules creates tracks for motor-mediated transport of synaptic cargo into the spine (Esteves da Silva et al., 2015). Based on recent data, it is interesting to speculate that actin reorganization to form a microtubule-capturing structure is a commonality between developmental and activity-dependent microtubule invasion processes. During major dendrite branching in *Drosophila* da neurons, extension of the tail of a subset of actin filaments toward the center of the dendrite growth cone is used to regulate the capture and guidance of polymerizing microtubules into filopodia (Yoong et al., 2020). In rodent hippocampal neuron cultures and slice cultures, spine activation leads to Cortactin-mediated projection of actin filaments into the main dendrite trunk from the base of the spine, and these filaments guide microtubules polymerizing along the main dendrite to turn into and invade the spine (Schatzle et al., 2018).

*Drosophila* da sensory neurons have been the major model used to study how and where microtubules are generated for late-stage dendrite branching processes. Local focal sites of microtubule generation at branchpoints contribute to invading microtubules (Ori-McKenney et al., 2012); additional sites within the arbor also contribute microtubules that polymerize along the main shaft and turn to invade nascent branches (Yalgin et al., 2015). In the mature stage, the branchpoint-associated sites continue to generate microtubules and are important for maintaining the minus-ends-out organization of the dendrites (Nguyen et al., 2014; Weiner et al., 2020). While it remains possible that there are changes in branchpoint site operation from the period of late-stage branching through into the mature neuron state, present data does not indicate that they are different.

Dendrites contain fragments of Golgi stacks named Golgi outposts, as show in rat hippocampal neurons and *Drosophila* neurons (Horton and Ehlers, 2003; Ori-McKenney et al., 2012). Initial studies in *Drosophila* da neuron dendrites colocalized MTOC sites with transgenic markers for Golgi (Ori-McKenney et al., 2012; Zhou et al., 2014; Yalgin et al., 2015). In several non-neuronal mammalian cell types Golgi stacks nucleate microtubules (Martin and Akhmanova, 2018; Akhmanova and Steinmetz, 2019; Valenzuela et al., 2020). For example, in human pigment epithelial cells, the Golgin GM130 recruits CG-NAP to the Golgi surface, which in turn brings in CDK5RAP2 and MYOMEGALIN to tether and activate γ-TuRC (Rivero et al., 2009; Wu et al., 2016). Golgi outposts also organize microtubules in the branches of rodent oligodendrocytes. Although in this case the microtubule generation is not γ-Tub dependent; instead, it is through a specialist tubulin polymerization promoting protein (TPPP)-mediated mechanism (Fu et al., 2019). Unidirectional microtubule generation was shown to be promoted from outposts in *Drosophila* sensory da neurons by Plp, Cnn, and GM130 (Ori-McKenney et al., 2012; Zhou et al., 2014; Yalgin et al., 2015; Figure 3B). Overall, these studies led to a model in which an outpost MTOC generates a unipolar train of microtubules which controls the local balance of anterograde and retrograde microtubules, and this activity alters the probability that a local nascent branch invaded and stabilized into a *bona fide* branch (Delandre et al., 2016). However, this model is not supported by all findings. In mature *Drosophila* da neurons, only a limited number of Golgi outposts in neurons were shown to associate with γ-Tub (Nguyen et al., 2014; Mukherjee et al., 2020; Weiner et al., 2020; Yang and Wildonger, 2020). In mature *Drosophila* da neurons, the main site of Golgi-mediated microtubule generation was shown to be from stacks in the soma, which generate a population of microtubules that exclusively invade the axon (Mukherjee et al., 2020; Figure 3B).

Therefore, there must be additional platforms for high order branch-related MTOC assembly. In both *Drosophila* da neurons and *C. elegans* PVD neurons, endosomes provide an alternative platform for γ-TuRC localization (Liang et al., 2020; Weiner et al., 2020). Recent studies in *Drosophila* da neurons examine this MTOC function in the mature neurons. Two intersecting sets of components are required for localization of γ-Tub at Rab5-positive endosomes at a branchpoint: the γ-TuRC-TPs Cnn and Plp, and members of the Wnt signaling pathway (Nye et al., 2020; Weiner et al., 2020). Disrupting the activity of several Wnt signaling proteins alters the overall balance of microtubule polarity in the dendrites (Weiner et al., 2020; Figure 3B). In dividing mammalian cells, such as HeLa cells, Wnt signaling pathway components Dishevelled segment polarity protein (Dvl) and Axin are localized to the centrosome, where Axin binds and recruits γ-Tub (Fumoto et al., 2009; Kikuchi et al., 2010; Ruan et al., 2012; Cervenka et al., 2016). In *Drosophila* da neuron dendrites, Dvl and Axin localize at branchpoint endosomes. Overall, Axin is the central scaffolding factor; it recruits both γ-Tub and Cnn to establish this site as an MTOC (Weiner et al., 2020).

## Neuron Diversification Involves Differential Regulation of MTOC Mechanisms

Neurons develop specific architectures to support their functional requirements; one way in which this manifests is in the organization of their microtubule cytoskeleton. For example, MTOC organization is notably different in early DRG compared with hippocampal neuron cultures (Mori et al., 2009; Stiess et al., 2010; Figure 1), and studies in *Drosophila* and *C. elegans* have shown how microtubule density and the localization of γ-Tub and microtubule minus-end foci differ between dendrite arbors of different neuron types (Yalgin et al., 2015; Delandre et al., 2016; Harterink et al., 2018; Mukherjee et al., 2020).

The stereotyped patterns of dendrite and axon arbors are genetically encoded by transcription factors (Jinushi-Nakao et al., 2007; Dong et al., 2015; Enriquez et al., 2015). One way by which these transcription factors regulate arbor patterning is through controlling the expression of cytoskeleton regulators including factors that control MTOC activity. This has been shown in *Drosophila* da neurons, which are excellent models in which to address how differentiation processes are modified to create neurons with distinct morphologies. They exist in four principal types named c1da–c4da in order of increasing complexity in their characteristic dendrite arbor shapes, and these characteristic shapes are defined through da neuron type-specific transcription factor codes (Dong et al., 2015). The c1da neuron-specific BTB-ZF (broad complex, tramtrack and bric à brac-zinc finger) family transcription factor Abrupt controls Cnn levels, then Cnn positions and orients microtubule generation events in the differentiating arbor at sites that include Golgi outposts. An interaction between this Cnn activity and Augmin activity sets the frequency at which polymerizing microtubules invade nascent branches (Yalgin et al., 2015). Because neuron morphogenesis is a compound process (Hassan and Hiesinger, 2015; Yoong et al., 2019), relatively small changes to a neuron morphogenetic program can translate into larger changes in final wiring pattern (Yalgin et al., 2015). In *Drosophila* da neurons, changing the frequency at which polymerizing microtubules invade nascent branches correlates with branch outgrowth, and ultimately with arbor final branch number (Ori-McKenney et al., 2012; Stewart et al., 2012; Yalgin et al., 2015).

Another example of transcription factor mediated regulation occurs at the dendrite tip MTOC in *Drosophila* da neurons. At this MTOC, the c4da neuron-specific EBF (Early B-cell factor) family transcription factor Knot regulates the position of microtubule generation events. In *knot* mutants the tip MTOC becomes disorganized; more microtubules are generated in the periphery of the dendritic growth cone and they polymerize in a retrograde direction rather than an anterograde direction. Knot-mediated regulation of the tip MTOC activity occurs in part through upregulating the expression of Myosin6. Ultimately, changing Knot and Myosin6 activity correlates with altered major branch frequency in the arbor (Yoong et al., 2020).

To fully understand the fundamental mechanisms that create form and function in nervous systems requires that investigators not only identify the components of the neuron differentiation process, but also understand the operational control mechanisms that direct and shape their usage. Understanding how diversity in MTOC organization arises between neuron types can be a powerful way to reveal operational controls over the neuron differentiation process at the systems level.

## Alternative Machineries for Generating Neuronal Microtubules

Additional γ-TuRC-independent mechanisms generate microtubules and position their minus-ends to shape dendrite and axon outgrowth, branching, and polarity organization. In *Drosophila* da neurons, Patronin binds to the minus-ends and promotes their polymerization. This allows the minus-ends to grow in an anterograde direction into dendrite branches to boost the minus-ends-out population in this compartment (Feng et al., 2019). Microtubule severing proteins such as Katanin and Spastin fragment pre-existing microtubules. This creates new local seeds and catalyzes microtubule formation (Vemu et al., 2018). In rodent hippocampal neurons and *Drosophila* da and motoneurons, the activity of these microtubule severing proteins shapes outgrowth and branching in both axon and dendrite compartments (Jinushi-Nakao et al., 2007; Yu et al., 2008; Qiang et al., 2010; Stewart et al., 2012; Mao et al., 2014).

While centrosomal and acentrosomal MTOC factors have been systematically examined in postmitotic neurons, it is likely that important non-canonical microtubule generation processes remain to be discovered. This is emphasized by the recent discovery of the microtubule generation activities of Sjögren’s syndrome nuclear autoantigen 1 (SSNA1). SSNA1 localizes at axon branchpoints in cultured rodent hippocampal neurons. *In vitro* it drives the forking of pre-existing microtubules to induce branch formation. These *in vitro* studies show that SSNA1 fibrils lie along the side of a microtubule, where they guide a subset of parental microtubule protofilaments to splay out. The splayed protofilaments seed a microtubule branch (Basnet et al., 2018).

A further potential new mechanism is based on how centrosomes increase in microtubule generation capacity at the onset of mitosis. Homotypic protein-protein interactions between scaffolding proteins (*Drosophila* Cnn or *C. elegans* SPD-5) cause these factors to concentrate from the cytoplasm into a non-membrane-bound compartment. This compartment captures and concentrates Tubulin from the surrounding environment to stimulate local microtubule production (Feng et al., 2017; Woodruff et al., 2017). Mammalian Tau is a neuronal candidate for this model of nucleation activity. In *in vitro* studies, Tau can transition into a similar compartment that captures Tubulin to stimulate local microtubule nucleation independently of γ-Tub. Moreover, it organizes the resultant microtubules to resemble their bundled organization in axons (Hernandez-Vega et al., 2017). Whether this process functions in *in vivo* remains to be determined.

## Future Challenges

The studies described here show how several distinct mechanisms for microtubule generation occur in the neurons. Nevertheless, as emphasized by the recent findings of TPPP (in oligodendrocytes) and SSNA1 (in neurons); (Basnet et al., 2018; Fu et al., 2019), it is likely that specialized and non-canonical microtubule generation processes remain to be discovered.

What structures enable γ-TuRC-TPs localization to create neuronal MTOCs? Recent studies in invertebrates have found endosomes are one platform upon which a dendrite MTOC can be established (Liang et al., 2020; Weiner et al., 2020). There is conflicting evidence whether Golgi outposts are another (Ori-McKenney et al., 2012; Nguyen et al., 2014; Zhou et al., 2014; Yalgin et al., 2015; Mukherjee et al., 2020; Weiner et al., 2020; Yang and Wildonger, 2020). Nevertheless, γ-Tub is found at many sites throughout the dendrite arbor in invertebrate and rodent neurons; do other γ-TuRC-TPs localization platforms exist?

Ultimately, mechanisms of γ-TuRC-TPs usage and positioning will be shaped by neuron type and differentiation stage. Just as neuron polarization mechanisms differ between neuron types due to intrinsic programming and interplay with the local environment (Yogev and Shen, 2017), the same is likely for neuron microtubule generation mechanisms—with an added critical dimension that the sites and mechanisms of microtubule generation shift as the neuron proceeds along its differentiation trajectory. Importantly, control mechanisms that regulate these critical transitions in MTOC mechanism are presently unknown; this key question is now opening for analysis. A further challenge is to consider how distinct neuronal MTOC mechanisms operate and interact at the systems level. The field will benefit from new generations of cell biologically informed computational models of differentiation to aid this (Goodhill, 2018). Crucially, understanding how individual microtubule generation mechanisms combine to delineate mature neuron function requires detailed long-term imaging of the cell biological events underlying arbor differentiation, with quantitative analyses of these events.

Neurons respond to injury with upregulation of microtubule generation in the axons and dendrites, as shown in *Drosophila* da neurons, *C. elegans* PLM neurons, and rodent intercostal nerves (Stone et al., 2010; Chen et al., 2012; Ghosh-Roy et al., 2012; Song et al., 2012; Kleele et al., 2014). One role of this is as a signal that upregulates neuroprotective programs, as demonstrated in *Drosophila* da neurons (Chen et al., 2012). In addition, damaged axon stumps form into a disorganized retraction bulb, which must then be converted into a functional growth cone to regrow. In rodent axon regeneration after spinal cord injury, mild pharmacological stabilization of axon tip microtubules helps to enhance this conversion (Hellal et al., 2011). A nuanced balance between dynamic and stable microtubules is required to stimulate axon regrowth (Blanquie and Bradke, 2018) and studies in *Drosophila* and *C. elegans* neurons suggest that injury-induced upregulation of microtubule dynamics helps prepare the local axon microtubule environment for this regrowth (Ghosh-Roy et al., 2012; Song et al., 2012). Beyond understanding differentiation, the discovery and elucidation of new neuronal microtubule nucleation pathways also provides potential targets for drug development to promote nervous system repair (Blanquie and Bradke, 2018).

In summary, an unfolding series of cell biological morphogenetic processes create final neuronal pattern (Hassan and Hiesinger, 2015; Yoong et al., 2019). In this review we have highlighted how molecularly distinct MTOC mechanisms create microtubules during these different stages of differentiation, and we have shown how temporal and spatial organization of these mechanisms are used to pattern and diversify dendrite and axon arbor wiring.

## Author Contributions

OW and AM: concept and wrote, and edited the manuscript. Both authors contributed to the article and approved the submitted version.

## Conflict of Interest

The authors declare that the research was conducted in the absence of any commercial or financial relationships that could be construed as a potential conflict of interest.
